# Associations between Parent–Child Nature Visits and Sleep, Physical Activity and Weight Status among Finnish 3–6-Year-Olds

**DOI:** 10.3390/ijerph182312426

**Published:** 2021-11-25

**Authors:** Juuli-Mari Kokkonen, Henna Vepsäläinen, Anna Abdollahi, Hanna Paasio, Samuli Ranta, Maijaliisa Erkkola, Eva Roos, Carola Ray

**Affiliations:** 1 Folkhälsan Research Center, Topeliuksenkatu 20, 00250 Helsinki, Finland; anna.abdollahi@helsinki.fi (A.A.); hanna.paasio@folkhalsan.fi (H.P.); eva.roos@folkhalsan.fi (E.R.); carola.ray@folkhalsan.fi (C.R.); 2 Department of Food and Nutrition, University of Helsinki, P.O. Box 66, 00014 Helsinki, Finland; henna.vepsalainen@helsinki.fi (H.V.); maijaliisa.erkkola@helsinki.fi (M.E.); 3 School of Applied Educational Science and Teacher Education, Philosophical Faculty, University of Eastern Finland, P.O. Box 111, 80101 Joensuu, Finland; samuli.ranta@uef.fi; 4 Department of Public Health, Clinicum, University of Helsinki, P.O. Box 20, 00014 Helsinki, Finland; 5 Department of Food Studies, Nutrition and Dietetics, Uppsala University, P.O. Box 560, 751 22 Uppsala, Sweden

**Keywords:** outdoor visits, sleep consistency, recreation, actigraph, overweight, young children

## Abstract

Nature visits and nature exposure have been shown to be favorably associated with children’s health and development, but the research regarding their associations with children’s lifestyle habits is limited. The current study aimed to investigate the associations between the frequency of parent–child nature visits and sleep, moderate-to-vigorous physical activity (MVPA) and weight status among three- to six-year-old Finnish preschoolers. Parents and their children (n = 864) participated in a cross-sectional DAGIS (increased health and wellbeing in preschools) study, which was conducted between 2015 and 2016 in Finland. In total, 798 parents answered a questionnaire on the frequency of parent–child nature visits, which also included questions on sociodemographic factors and their children’s sleep habits. Parents also reported children’s bedtimes and wake-up times and children wore an accelerometer for seven days. Trained researchers measured children’s weight and height. Linear and logistic regression analyses were conducted. More frequent parent–child nature visits were associated with children’s longer sleep duration at night, higher amounts of MVPA outside preschool time and, among girls, good sleep consistency. The frequency of parent–child nature visits was not significantly associated with whether children were overweight or obese or not. Promoting parent–child nature visits could be a cost-effective way to increase young children’s MVPA and enhance night-time sleep.

## 1. Introduction

Nature may offer an affordable environment for families with children to engage in physical activity and recreational activities. Parents act as enablers of young children’s nature visits [1,2], and nature experiences in childhood are important, as they may predict nature exposure and physical activity in a natural environment as an adult [3,4]. Children tend to spend more time in built environments [5] or watching different screens [2] than in nature, despite the benefits that nature exposure could provide. Nature exposure has been shown to have favorable associations with children’s health and development, such as good physical fitness [6] and better mental well-being, as well as an inverse association with behavioral problems and symptoms of attention-deficit hyperactivity disorder (ADHD) [6,7]. In addition, natural environments might encourage children to participate more in imaginative play compared to built environments without greenery [6].

Limited park accessibility has been associated with sleep problems among primary school children and adolescents [8], although lack of greenery in the residential area has not shown similar associations [7]. A study conducted in Sweden showed that outdoor play in a good-sized outdoor environment with well-integrated greenery in the play areas of an early childhood education and care (ECEC) center was associated with a longer night’s sleep in preschool children [9], while in other studies the association with outdoor play has not been significant [10,11]. The relationship between sleep and nature exposure has not been studied much, and what research has been conducted has mainly focused on adults [7]. Global sleep recommendations suggest 10 to 13 h of sleep daily for three- to five-year-old children [12]. Sufficient sleep is important for children, as shorter sleep duration has been associated with poorer growth and emotional regulation [13], poorer eating habits [14] and obesity [13,15], while longer sleep duration has been associated with higher amounts of physical activity in some studies [13]. There is an evident lack of research into nature visits and preschool children’s sleep, and, given the health benefits of sufficient sleep, conducting research on this subject is clearly valuable.

Outdoor play [16] and access to green spaces (e.g., urban parks and playground areas) for preschool children [6,17], and, among adolescents, higher maternal participation in visits to nature (e.g., forests or lakes) or other green spaces (e.g., parks) [18], have been shown to have positive associations with the overall physical activity of children. The association between nature visits and the amount of moderate-to-vigorous physical activity (MVPA) has mainly been found in adolescents [7,19] and not in preschool children [20,21]. Global physical activity (PA) guidelines for children under five years of age recommend three hours of PA per day with at least 60 min spent engaging in MVPA [12]. MVPA is associated with many positive health indicators in children, such as better motor development and skeletal health, as well as healthy weight status in some studies [22].

Higher amounts of outdoor play have been associated with a lower body mass index (BMI) among preschool children [23] and adolescents [19]. In addition to outdoor play, greener neighborhoods have been associated with the healthier weight status of preschool children [6,24] and adolescents [7], and a lower risk of these children becoming overweight [25] or having higher BMI z-scores over time [26]. However, there may be differences depending on the type of green area. Children aged two to seventeen who live within a close distance to parks with playgrounds were more likely to have a healthy weight status [27], although the association has not always been detected [17]. Moreover, parks with natural greenery, such as wooded or water areas, have not been associated with the weight of two- to seventeen-year-old children [27]. Even though some studies have investigated the role of the neighborhood and outdoor play in health and development, more research is needed, especially into preschool children and their families. It is important and valuable to investigate this association, because it will help identify affordable and feasible means to prevent childhood obesity, which is a growing global public health problem [28] that is associated with several comorbidities, such as metabolic risk factors, asthma and ADHD [29].

Previous studies have shown some associations between nature visits or green space exposure and children’s sleep [8,9], PA [6,7,16,18] and weight status [6,7,17,19,23,24,25], but studies including preschool children are still scarce. Studies involving preschool children should be conducted as well, as nature exposure in childhood may predict nature exposure in adulthood [3,4]. There is also a lack of research investigating the associations between nature visits and preschool children’s sleep, MVPA and weight status in the same study. The aim of the current study was to investigate the associations between the frequency of parent–child nature visits and sleep, MVPA and weight status among Finnish preschool children aged three to six years. The research questions were: (1) Is the frequency of parent–child nature visits associated with night-time sleep duration and sleep consistency in children? (2) Is the frequency of parent–child nature visits associated with the amount of children’s MVPA outside preschool time? (3) Is the frequency of parent–child nature visits associated with children’s weight status? We also examined whether there were differences in these associations between genders. In this study, parent–child nature visits are defined as visits to natural environments (e.g., forests or lakes) with an adult in the same household, and can consist of any kind of outdoor activity (e.g., outdoor or nature play or walking).

## 2. Materials and Methods

### 2.1. Study Design and Participants

This study utilizes data from a cross-sectional study conducted in 2015 to 2016 as a part of the DAGIS (increased health and wellbeing in preschools) study. The cross-sectional study aimed to investigate socioeconomic differences in children’s energy balance-related behaviors. The study included 864 children aged three to six years and their parents from eight municipalities and 66 ECEC centers in southern and western Finland. More details about the cross-sectional DAGIS study can be found elsewhere [30]. The sample size of the current study was 798, and consisted of children and their parents who had answered the question on the frequency of parent–child nature visits. The University of Helsinki Ethical Review Board in the Humanities and Social and Behavioral Sciences has given a statement of ethical approval for the DAGIS cross-sectional study (6/2015).

### 2.2. Measures

The variables measured in this study were children’s sleep duration and sleep consistency, children’s MVPA outside preschool time, children’s weight status and the frequency of parent–child nature visits.

Children’s sleep duration at night was calculated from a seven-day sedentary behavior diary where parents had reported their child’s bedtimes and wake-up times. Data from at least three weekday nights (Sunday–Thursday) and one weekend night (Friday–Saturday) were required to be included in the analyses. The total sleep duration mean variable was conducted by summing up the mean values for weekdays and weekend days: (sleep duration on weekdays × 5 + sleep duration on weekend days × 2)/7.

Parents answered questions about their child’s sleep habits. The questions were based on the children’s Sleep Habits Questionnaire [31]. A mean variable of sleep consistency (Cronbach’s alpha 0.693) was calculated based on three items (A, B, C) of the questionnaire. This method is based on a previous study [32]. From their last typical week, parents were asked to recall how many times their “A. child went to bed at the same time at night? B. child slept the right amount? C. child slept about the same amount each day?”. The answer options were: 1 = never, 2 = 1–2 times per week, 3 = 3–4 times per week, 4 = 5–6 times per week or 5 = daily. Due to a non-normal distribution, the mean variable was divided into tertiles, of which the middle and the highest tertiles were combined. The lowest tertile represented poor sleep consistency and the middle and highest tertiles represented good sleep consistency.

Children were instructed to wear Actigraph wGT3X-BT (Pensacola, FL, USA) accelerometers for seven consecutive days, 24 h per day. In addition, parents reported their child’s preschool hours in a diary. The accelerometers were fitted around children’s waists in preschool by the research assistants and parents received written instructions regarding the use of the accelerometer. A 15 s epoch length was used and periods of 10 minutes or more of consecutive zeroes were set as non-wearing time [33]. Evenson cut-points were applied to define the moderate and vigorous intensity PA moments in the data [34]. For the MVPA data to be included in the analyses, there had to be data from at least three weekdays with preschool attendance and at least one weekend day. A valid day was defined as at least 600 min of awake wearing time. To calculate the average MVPA minutes per hour, the MVPA variable was divided by the wearing time of the accelerometer and multiplied by 60 min.

Trained researchers measured children’s weight using CAS portable bench scales (CAS PB-100/200) and height using stadiometers (SECA 217). BMI was calculated as body weight (kg)/height^2^ (m). Whether children were overweight, obese or neither was defined by using cut-offs from the Finnish references for BMI-for-age according to Saari et al. [35], as well as using the age- and gender-specific BMI cut-offs of the International Obesity Task Force (IOTF) criteria [36]. According to these cut-offs, two dichotomous variables of children’s weight status were formed (0 = underweight or normal weight, 1 = overweight or obese).

The frequency of parent–child nature visits was determined by a question which asked: “How often does your child go to the nature/forest with at least one of the adults in the family?” Parent–child nature visits refer to visits to natural environments, such as forests or lakes. A family’s own yard, built playgrounds or built parks were not included in this question. The answer options were recoded based on how many times such nature visits occurred per week: 0 (less than once a month), 0.5 (1–3 times per month), 1.5 (1–2 times per week), 3.5 (3–4 times per week), 5.5 (5–6 times per week) and 7 (daily).

### 2.3. Covariates

The respondent of the questionnaire, the child’s age and gender, the highest educational level of parents and the season of measurement were the covariates in the analyses. The respondent of the questionnaire was the mother or the father of the child, or another guardian. The respondent parent or guardian reported the child’s gender and birth date (from which the child’s age was calculated). The respondent parent or guardian reported their own and their cohabiting partner’s highest educational level from six options. The highest parental educational level in each family was divided into three categories (low = high school or vocational school graduate or lower; middle = Bachelor’s degree or equivalent; high = Master’s degree or higher). The season of measurement had three categories (September to October, November to December and January to April).

### 2.4. Statistical Analyses

The gender differences between the participant characteristics were analyzed using chi-square tests for categorical variables, *t*-tests for normally distributed variables and Mann–Whitney tests for non-normal distributed variables. Correlations between the variables were determined using Spearman’s correlation tests. Linear regression analyses were conducted to investigate the associations between the frequency of parent–child nature visits and child’s sleep duration and MVPA. Logistic regression analyses were conducted to investigate the associations between the frequency of parent–child nature visits and children’s sleep consistency and whether they were overweight or obese. All analyses were adjusted according to the respondent of the questionnaire, the child’s age and gender, the highest educational level of parents and the season of measurement. There was no multicollinearity detected (the variance inflation factor values, or VIF, were <2) in any of the regression analyses that were conducted. Possible differences in the associations between genders were observed with interaction terms in the models. The level of significance was established at *p* < 0.05. The statistical analyses were performed by using IBM SPSS Statistics 28 software.

## 3. Results

The descriptive statistics are presented in Table 1. The question on the frequency of parent–child nature visits was answered by 798 parents and the respondents were mainly mothers (88%). The children’s mean age was 4.7 (±0.9) years. The mean night-time sleep duration of the children was 10 h and 22 min (±0:33) and they spent 5.2 (±1.7) min/h engaged in MVPA on average. Boys spent more time engaged in MVPA than girls (*p* < 0.001). Of the participant children, 12–15% were overweight or obese. According to the cut-offs from the Finnish references for BMI-for-age, there were more overweight or obese boys than girls among the participants (*p* < 0.001).

### 3.1. Associations between the Frequency of Parent–Child Nature Visits and Children’s Sleep Habits

Table 2 shows the results of the linear regression analysis of the association between the frequency of parent–child nature visits and children’s sleep duration. The model found a statistically significant association between more frequent parent–child nature visits and longer sleep duration in children. The significant association remained after adjustments were made. Table 3 displays the results of the logistic regression analysis of the association between the frequency of parent–child nature visits and children’s sleep consistency. A significant interaction was found for good sleep consistency and, therefore, the association was examined separately for girls and boys. Girls who visited nature more frequently with a parent were more likely to have good sleep consistency than girls who rarely visited nature with a parent. No significant associations were found with this variable for boys.

### 3.2. Association between the Frequency of Parent–Child Nature Visits and the Amount of Children’s MVPA Outside Preschool Time

Table 2 shows the results of the linear regression analysis of the association between the frequency of parent–child nature visits and children’s MVPA outside preschool time. There were significant associations in both the unadjusted and adjusted models. More frequent visits to nature with a parent were associated with more MVPA outside of preschool.

### 3.3. Association between the Frequency of Parent–Child Nature Visits and Children’s Overweight or Obesity Classification

Table 4 shows the results of the logistic regression analysis of the association between the frequency of parent–child nature visits and children’s overweight or obesity BMI classification. There were no significant associations between these variables.

## 4. Discussion

The aim of this study was to investigate the associations between the frequency of parent–child nature visits and sleep, MVPA and weight status among Finnish preschool children aged three to six years. The results showed that more frequent parent–child nature visits were associated with higher amounts of MVPA outside preschool time, longer sleep duration at night and, among girls, good sleep consistency. The results were also significant after the models were adjusted for confounding factors. There was no significant association between the frequency of parent–child nature visits and children’s overweight or obesity classification.

Spending time in nature has previously been shown to have a favorable association with preschool children’s sleep duration [9], which is in line with our results. However, there is also evidence that no significant associations have been found [7,10,11]. Nature exposure and its association with fewer hyperactivity symptoms [6,7], and in some studies less stress [6], might partly explain the positive association. Spending time outdoors might provide an outlet for pent-up energy as children are more active outdoors than indoors [7,19], which may help children tire in the evening. Further studies are needed to verify these associations and to elucidate the possible mechanisms driving them. In addition to sleep duration, there was a positive association between sleep consistency and the frequency of parent–child nature visits among girls. Sleep consistency in our study consisted of going to bed at the same time, sleeping the same amount and sleeping the right amount. The lack of differences between boys’ and girls’ sleep consistency or the frequency of parent–child nature visits provided no explanation for the result. Moreover, the children in our study were young, which means that they likely had consistent sleep habits set by their parents regardless of the child’s gender. It should be noted that there are several factors which can affect sleep consistency in children, such as medical problems or other home factors related to sleep habits, which we did not measure in our study. Future studies could focus on investigating the gender differences and the associations between parent–child nature visits and children’s sleep consistency and sleep quality (e.g., sleep latency and night wakings) more comprehensively.

Previous research has shown that outdoor play [20] or park visits with a parent [21] and the amount of MVPA of preschool children were not associated, which is opposite to our results. However, similar association to ours have previously been found in adolescents [7,19]. In previous studies, the natural environment might have been defined differently compared to our study. In our study, the environment was described as nature or forest. Finnish forests are easily accessible and free for all inhabitants, which may not be the case in all countries. Typically, Finnish forests have different landscapes and an uneven surface, which can provide an environment where children might engage more in MVPA. Children can also be more encouraged to participate in imaginative and sensory play in natural areas compared to built or flat play areas, as previously stated [6], which can lead to more intensive physical activity. Future studies could compare differences between parent–child nature visits in different outdoor areas and children’s MVPA in order to obtain a better understanding of places where children are most active. This could be beneficial information when planning family-based interventions for promoting PA.

Whether children were overweight or obese was not associated with the frequency of parent–child nature visits in our study. Previously, the association between lower BMI and outdoor play has been found mainly among adolescents [19]. Moreover, a neighborhood’s greenness has been associated with healthier weight status in children of various ages [6,7,24,25]. There is also evidence that living near a playground is associated with children’s healthy weight status, whereas a close distance to natural environments is not [27]. It could be that parents take their child on nature visits whether the child is overweight or not, compared to playgrounds, where the parents might, for example, find that the equipment is too difficult for their child to use if they are overweight. Furthermore, overweight children attend sports clubs less than their normal weight peers [37]. This could indicate that parent–child nature visits can be a way to promote children’s PA despite the child’s weight status. Nevertheless, we cannot confirm whether more frequent nature visits are not associated with child’s healthier weight status or if the frequency of parent–child nature visits is the same despite the child’s weight status. Therefore, it would be beneficial to study the association using a longitudinal design, which would help to detect changes over time. It should also be noted that there are many other energy balance-related behaviors that can influence children’s BMI, such as nutrition and other PA activities. Nature visits are only one form of activity, and they may not be independently associated with a healthier weight status in children.

Our study has several strengths. Children’s MVPA was measured using accelerometers and children’s weight and height were measured by trained researchers, which means that we have collected an array of objective data. To obtain a more comprehensive view on the association with sleep, we used two measurements: night-time sleep duration and sleep consistency. Another strength was that the participants were recruited from different areas in Finland and there were participants both from rural and urban areas. Our study also provides new information on the associations between parent–child nature visits and preschool children’s lifestyle habits, which is a fairly unexplored topic. To the best of our knowledge, this is the first time that associations between nature visits and children’s sleep, MVPA and weight status are explored in the same study.

The following limitations should be considered when interpreting the results of our study. The study design was cross-sectional and, therefore, no cause-and-effect relationships can be determined. There were also some limitations concerning the variables. Children’s sleep duration was calculated from parent-reported bedtime and wake-up times and no objectively measured data (e.g., accelerometer data) was used. According to previous studies, there are differences in children’s sleep duration depending on whether it is reported by the parent or if it is measured with an accelerometer [38,39,40]. In our study, parents might have also answered in a way that they perceived to be socially acceptable, which can lead to response bias. Therefore, in future studies, it would be beneficial to use objectively measured data on children’s sleep duration. Children’s sleep consistency was also measured with questions answered by their parents. Even though the mean variable of sleep consistency was formed based on a previous study [32], we cannot confirm that it measures sleep consistency properly. Furthermore, there might be other more suitable ways to divide the data into good and poor sleep consistency, rather than dichotomizing it using tertiles, as we have here.

Limitations also extend to the measurement of weight status. Even though BMI is a widely used measurement to class individuals as overweight and obese, it is not simple to determine whether children are overweight, for example, due to age-related differences and varying cut-offs for the definition of overweight [41]. Therefore, it can be difficult to compare results from different studies. In order to provide comparable results, we used both national [35] and international [36] age- and gender-specific BMI cut-offs, both of which showed similar results with no significant association between parent–child nature visits and overweight or obesity classifications in children. However, according to the power analyses, a larger sample size would have been needed to properly conduct these logistic regression analyses in order to obtain statistically significant results, as the value of the estimates was small and had a large mean error.

It should be noted that there can be other factors which might affect the results of our study. For example, the time children have spent in ECEC centers during the day, screen time and time spent in nature could have made a difference to the frequency of parent–child nature visits, as well as sleep duration, sleep consistency, MVPA levels, weight status and their associations. These other factors and their interrelations should be taken into consideration in future studies.

Despite these limitations, our study provides important and new information on the associations between parent–child nature visits and children’s lifestyle habits. Further studies are needed in order to confirm these associations and objective methods should be used. Moreover, conducting longitudinal studies would provide more comprehensive results.

## 5. Conclusions

The results suggest that more frequent parent–child nature visits are associated with three- to six-year-old Finnish children’s longer sleep duration at night, higher levels of MVPA outside preschool time and, among girls, good sleep consistency. The frequency of parent–child nature visits was not associated with whether children were overweight or obese or not. According to these results, promoting parent–child nature visits could be a cost-effective way to increase young children’s MVPA levels and enhance night-time sleep.

This study highlights the importance of nature for children’s health. To enable and increase family’s nature visits in both urban and rural residential areas, accessible nature areas should be available within close distances to homes in order to ensure access for all and to limit possible barriers (e.g., lack of time or transport difficulties) to visiting nature. Collaboration with municipalities and organizations would be beneficial in order to inform the public about these free recreational outdoor opportunities and to plan suitable family-based nature activities.

## Figures and Tables

**Table 1:** Descriptive statistics of 798 participants.

Variables	All (n = 716–798)		Girls (n = 345–384)		Boys (n = 371–414)		
	Mean ± SD	n (%)	Mean ± SD	n (%)	Mean ± SD	n (%)	*p*-Value
Frequency of parent–child nature visits, times per week (range 0–7) ^1^	1.3 ± 1.5	798	1.2 ± 1.4	384	1.3 ± 1.5	414	0.254
Season of measurement ^2^							0.645
September–October		350 (44)		169 (44)		181 (44)	
November–December		287 (36)		133 (35)		154 (37)	
January–April		161 (20)		82 (21)		79 (19)	
**Children**							
Age (years) ^3^	4.7 ± 0.9	798	4.7 ± 0.9	384	4.8 ±0.9	414	0.335
Sleep duration (h:min/night) ^3^	10:22 ± 0:33	745	10:24 ± 0:33	361	10:20 ± 0:32	384	0.094
Sleep consistency ^2,4^							
Good sleep consistency		616 (78)		297 (78)		319 (77)	0.81
Poor sleep consistency		178 (22)		84 (22)		94 (23)	
MVPA outside preschool time (min/h) ^1^	5.2 ± 1.7	716	4.8 ± 1.5	345	5.5 ± 1.8	371	**<0.001**
Finnish references for BMI cut-offs ^2,5^							
Underweight or normal weight		633 (85)		331 (90)		302 (79)	**<0.001**
Overweight or obese		116 (15)		36 (10)		80 (21)	
IOTF criteria for BMI cut-offs ^2,6^							
Underweight or normal weight		660 (88)		323 (88)		337 (88)	0.93
Overweight or obese		89 (12)		44 (12)		45 (12)	
**Parent**							
Respondent ^2^							0.649
Mother		696 (87)		336 (87)		360 (87)	
Father		95 (12)		46 (12)		49 (12)	
Other guardian		4 (1)		1 (1)		3 (1)	
Highest parental educational level ^2^							
Low (≤high school level education)		179 (23)		86 (23)		93 (23)	0.117
Middle (Bachelor’s degree or equivalent)		334 (42)		173 (45)		161 (39)	
High (≥Master’s degree)		281 (35)		122 (32)		159 (39)	

Abbreviations: SD = standard deviation; MVPA = moderate-to-vigorous physical activity; BMI = body mass index; IOTF = International Obesity Task Force. The gender differences between the participant characteristics were analyzed using: ^1^ Mann–Whitney test for non-normal distributed variables; ^2^ Chi-square test for categorical variables; and ^3^ *t*-test for normally distributed continuous variables. ^4^ A mean variable of three items (in the last typical week: A. child went to bed at the same time at night; B. child slept the right amount; C. child slept about the same amount each day) dichotomized into good (two highest tertiles, range 4.00–5.00) and poor sleep consistency (the lowest tertile, range 0–3.99). ^5^ According to the Finnish references for BMI-for-age [35]. ^6^ According to the age- and gender-specific BMI cut-offs of the International Obesity Task Force criteria [36]. Statistically significant differences (*p* < 0.05) are presented as bold *p*-values.

**Table 2:** Linear regression analyses of the associations between the frequency of parent–child nature visits per week and children’s sleep duration and MVPA outside preschool time.

	Sleep Duration (h/per Day) (n = 740–745)				MVPA Outside Preschool Time (min/h) (n = 710–716)			
	Unadjusted		Adjusted ^1^		Unadjusted		Adjusted ^1^	
	β	95% CI	β	95% CI	β	95% CI	β	95% CI
Frequency of parent–child nature visits, times per week (range 0–7)	0.08 *	0.01–0.06	0.08 *	0.01–0.06	0.11 **	0.04–0.22	0.10 **	0.03–0.21

Abbreviations: CI = confidence interval; MVPA = moderate-to-vigorous physical activity. ** *p* < 0.01; * *p* < 0.05. ^1^ Adjusted for the respondent of the questionnaire, child’s age, child’s gender, highest educational level of parents and the season of measurement.

**Table 3:** Logistic regression analyses of the associations between the frequency of parent–child nature visits per week and children’s good sleep consistency.

	Good Sleep Consistency ^1^							
	Girls (n = 377–381)				Boys (n = 410–413)			
	Unadjusted		Adjusted ^2^		Unadjusted		Adjusted ^2^	
	OR	95% CI	OR	95% CI	OR	95% CI	OR	95% CI
Frequency of parent–child nature visits, times per week (range 0–7)	1.31 *	1.04–1.64	1.28 *	1.01–1.61	0.98	0.85–1.14	0.98	0.85–1.14

Abbreviations: OR = odds ratio; CI = confidence interval. * *p* < 0.05. ^1^ A mean variable of three items (in the last typical week: A. child went to bed at the same time at night; B. child slept the right amount; C. child slept about the same amount each day) dichotomized into good (two highest tertiles, range 4.00–5.00) and poor sleep consistency (the lowest tertile, range 0–3.99). ^2^ Adjusted for the respondent of the questionnaire, child’s age, highest educational level of parents and the season of measurement.

**Table 4:** Logistic regression analyses of the associations between the frequency of parent–child nature visits per week and children’s overweight or obesity BMI classification.

	Overweight or Obesity According to the Finnish References (n = 742–749) ^1^				Overweight or Obesity According to the IOTF Criteria (n = 742–749) ^2^			
	Unadjusted		Adjusted ^3^		Unadjusted		Adjusted ^3^	
	OR	95% CI	OR	95% CI	OR	95% CI	OR	95% CI
Frequency of parent–child nature visits, times per week (range 0–7)	1.03	0.90–1.17	1.00	0.87–1.15	1.05	0.90–1.21	1.03	0.88–1.19

Abbreviations: OR = odds ratio; CI = confidence interval; IOTF = International Obesity Task Force. ^1^ According to the Finnish references for BMI-for-age [35]. ^2^ According to the age- and gender-specific BMI cut-offs of the International Obesity Task Force criteria [36]. ^3^ Adjusted for the respondent of the questionnaire, child’s age, child’s gender, highest educational level of parents and the season of measurement.

## Data Availability

For questions concerning data availability, please contact Eva Roos.
